# Pharmacokinetic and pharmacodynamic properties of the new AEDs: A review article

**Published:** 2013

**Authors:** Majid Ghaffarpour, Hossein Pakdaman, Mohammad Hossein Harirchian, Hossein-Ali Ghelichnia Omrani, Mojdeh Ghabaee, Babak Zamani, Parviz Bahrami, Bahaadin Siroos

**Affiliations:** 1 Professor, Department of Neurology, Imam Khomeini Hospital, Iranian Center of Neurological Research, Tehran University of Medical Sciences, Tehran, Iran; 2 Professor, Department of Neurology, Shahid Beheshti University of Medical Sciences, Tehran, Iran; 3 Professor, Department of Neurology, Imam Khomeini Hospital, Iranian Center of Neurological Research, Tehran University of Medical Sciences, Tehran, Iran; 4 Assistant Professor, Department of Neurology, Imam Khomeini Hospital, Iranian Center of Neurological Research, Tehran University of Medical Sciences, Tehran, Iran; 5 Associate Professor, Department of Neurology, Imam Khomeini Hospital, Iranian Center of Neurological Research, Tehran University of Medical Sciences, Tehran, Iran; 6 Assistant Professor, Rasool-e Akram Hospital, Iran University of Medical Sciences, Tehran, Iran; 7 Associate Professor, Department of Neurology, Lorestan University of Medical Sciences, Lorestan, Iran; 8 Resident, Department of Neurology, Imam Khomeini Hospital, Iranian Center of Neurological Research, Tehran University of Medical Sciences, Tehran, Iran

**Keywords:** Epilepsy, Seizure, Pharmacokinetics, New Generation, Antiepileptic Drugs

## Abstract

The new-AEDs, whose developments were motivated following the discovery of the valproate and its marketing in the U.S in 1978, have presented more therapeutic options. There are approximately twenty four FDA-approved antiepileptic drugs for use in patients with epilepsy, five of which were identified and have come on to the market between 2009 and 2012. The new-AEDs are of interest, not due to their efficacy, but rather owing to better tolerance, favorable pharmacokinetic profile, fewer interactions, and in some instances, lesser protein binding. No standard AED or those in developing have all properties of an ideal antiepileptic drug, thus to achieve desirable outcome, physicians should be aware of pharmacokinetics (PKs) and pharmacodynamics (PDs) of drugs. This review describes briefly the major features of the new AEDs.

## Introduction

The interaction between drugs and their specific target molecules produces beneficial and adverse effects (AEs) of any treatment. The events occurring between administration of a drug and production of its effects can be divided into two components, the PKs and the PDs. The processes that determine drug delivery (absorption, bioavailability, distribution) to and removal (metabolism and elimination) from molecular targets are termed PKs. The processes that determine variability in drug actions despite equal delivery to target molecules (effector sites) or the relationship between concentration and effects are named PDs. The new-AEDs are not necessarily more effective than the traditional drugs, but have special PKs and PDs advantages that create additional and sometimes better options for treatment. These drugs have lower protein binding with the exception of fosphenytion, tiagabine, and ezogabine (Table 1), which are better tolerated, have fewer interactions, and usually fewer adverse effects.

**Table 1:** Grouping of the AEDs

Old AEDs			New-AEDs		

1993-2005			2009-2011		

	PB%		PB%		PB%
Phenobarbital	45	Felbamate	25	Vigabatrin	< 5
Phenytoin	90	Gabapentin	< 5	Clobazam	85
Clonazepam	85	Lamotrigine	55	Rufinamide	34-26
Diazepam	99	Levetiracetam	< 10	Lacosamide	< 15
Lorazepam	90	Tiagabine	96	Ezogabine	80
Ethosuximide	< 5	Pregabalin	< 5	Oxcarbazepine	40-60
Primidone	< 20	Topiramate	15	Eslicarbazepine acetate	30
Carbamazepine	75	Zonisamide	55	10-hydroxy-carbazepine	40
Valproate	70-93	Fosphenytoin	90-99		

Abbreviations: AAN: American Academy of Neurology, AEDs: Antiepileptic Drugs, AEs: Adverse Effects, EPs: Action Potenitals, ESM: Ethosuximide, GI: Gastrointestinal, LGS: Lennox-Gastaut syndrome, PGTCS: Primary Generalized Tonic Clinic Seizures, SE: Status Epilepticus, SGTCS: Secondary Generalized Tonic Clinic Seizures.

In addition, of the new AEDs only felbamate, oxcarbazepine, and topiramate are known to reduce the effectiveness of hormonal contraception.

Renal function has an indirect effect on the concentration of the commonly used traditional AEDs, whereas some newer drugs such as LEV, VGB, GBP, and PGB are eliminated by kidneys, requiring dosage adjustment in patients with renal failure.

Development of several drugs and three new trends (use of pharmacogenetics to predict serious side effects; e.g., strong association of Steven-Johnson syndrome with HLA-B 1502 in Southeast Asians and HLA-A 3101 in Europeans, generic substitution to brand of AEDs, and the FDA warning of the suicide risk of the entire class of AEDs) have helped physicians gain much knowledge about treatment and makes choosing the optional agent for individual patients a challenge. Although, FDA has supported bioequivalence of approved brand-name and generic AEDs and suggested that generic drugs may be safely interchanged with brand-name AEDs, neurologists and epilepsy advocacy groups have noted breakthrough seizures following switching brand-name drugs to generic AEDs. This issue is a matter of debate and the final answer is yet to be determined.

**Table 2:** Introduction of old and new AEDs

The older generation		New AEDs	

Name	Time of USA approval	Name	Time of USA approval
Bromides	1857*	FBM, GBP	1993
PHB	1920s-1940*	LTG	1994
PHT	1953 (FDA approved) -1938*	TGB, TPM	1997
ESM	1960	LEV	1999
CBZ	1974	OXC, ZNS	2000
		PGB	2005
		LCM	2009
		VGB**	2009
VPA	1978	RFN	2010
		CBM	2011
		EZG	2013

* Indicates time of development

** First was approved in Europe in 1989

CBM: clobazam, CBZ: carbamazepine, ESM: ethosuximide, EZG: ezogabine, FBM: felbamate, LCM: Lacosamide, LEV: levetiracetam, OXC: oxcarbazepine, PGB: pregabaline, PHB: phenobarbital, PHT: phenytoin, RFN: Rufinamide, TGB: tiagabine, TPM: topiramate, VGB: vigabatrin, VPA: valproic acid, ZNC: zonisamide

Most AEDs exert their effects via mechanisms that either diminish neuronal excitability or enhance neuronal inhibition by blocking Na+ channels or T-type Ca++ channels and enhancing GABA effects. At least two new antiepileptic drugs have novel mechanisms of action that differ from others; levetiracetam that binds to synaptic vesicle glycoprotein 2A (SV2A) and ezogabine, a unique drug that reduces neuronal excitability by enhancing K+ currents on the presynaptic membrane in the excitatory synapses. Physicians should use new AEDs correctly and professionally. The UK National Institute for Clinical Excellence (NICE) recommended that new AEDs be considered within their licensed indications (Box 1).

**Box 1**. Licensed indications for use of New AEDs

- Established drugs (typically CBZ or VPA) have failed
- An older drug could interact with other medications (including OCPs)
- The most appropriate older drugs are contraindicated
- The older drugs are already known to be poorly tolerated
- The patient is a women of childbearing potential (although the safety of new ADEs in pregnancy remain unclear)

### 1- Felbamate (Felbatol)

FBM (2-phenyl-1,3-propanediol dicarbamate) is a potent blocker of NMDA receptors and voltage-gated Ca-channels. It also modulates Na-channel conductance. It is 25% bound to plasma protein and is metabolized in liver. Its elimination half-life is 15-30 hours, but decreases to 14 hours when combined with enzyme inducers.

Felbamate is effective in partial and generalized seizures and reduces atonic seizures and improves the life of children with LGS. In July 1993 the FDA granted approval of felbamate for both monotherapy and add-on drug. However, a warning was added in 1994, because of reports of aplastic anemia and hepatitis. As a result, this drug should be reserved for those patients resistant to other effective treatments. Its dose in adults is 1800 to 4800 mg/day. In children doses of 15-45 mg/kg have been used. This drug increases PHT and VPA levels, but decreases the level of carbamazepine. It is prepared by 400 and 600 mg tablets and 600 mg/5 ml suspension.

### 2-Gabapentin (GBP: Neurontin)

Gabapentin is structurally similar to GABA and was approved for the treatment of partial and SGTCSs, though it is thought to have relatively low anticonvulsant potency.^1^

GBP, though designed as a simple analogue of GABA (GABA_A_ inhibitor), owes its antiepileptic effect mainly to an action on P/Q type Ca-channels, that reduces Ca^++^ entry into the nerve terminals and thereby reducing release of neurotransmitters and modulators. This channel has low activation threshold and becomes inactivated slowly.

It binds the alpha-2 delta subunit of Ca-channels in the cerebral cortex, hippocampus, and spinal cord, reducing influx of Ca^++^ at nerve terminals, in turn decreasing excitatory neurotransmitters’ discharge such as glutamate, substance-p, and noradrenaline, but does not block Ca-channels.^2–3^

Its absorption depends on a saturable active L-aminoacid transporter, thus unlike other new AEDs, it has poor availability (< 60%), even lower (10 to 35%) at doses higher than 1200 mg.

Gabapentin neither binds to plasma protein nor is metabolized in liver. It is excreted unchanged in urine, with elimination half-life of 5-9 hours. In addition, it does not induce hepatic enzymes. It has a lack of drug interaction, lack of protein binding, and is eliminated only by kidneys. Therefore, gabapentin is a useful drug in patients with hepatic and renal disease, but requires dosage adjustment according to creatinine clearance.^2–5^ Furthermore, its good tolerability may be a particularly important advantage in older people with epilepsy.

Gabapentin, like levetiracetam, ethosuximide, topiramate, vigabatrin, felbamate, and to some extent lamotrigine or phenobarbital, is removed during standard 4-hour hemodialysis by 50%, requiring supplement dose after hemodialysis.

It is contraindicated in patients with known hypersensitivity, pancreatitis, and galactosemia. Gabapentin capsules contain lactose, thus blood glucose should be monitored more frequently in patients with diabetes mellitus. This drug does not affect metabolism of other drugs, and is therefore an ideal drug as add-on-therapy.

Although it is generally well tolerated, side effects such as somnolence, ataxia, and dizziness are common. Fatigue, impotence, nausea, and slurred speech, which are usually mild, were also reported. Few children may exhibit behavioral changes, including hyperactivity and agitation. No significant idiosyncratic reaction or systemic AEs have been detected.^6^

### 3-Lamotrigine (LTG: Lamictal)

Lamotrigine is a broad spectrum AED that was first discovered when phenytoin derivatives with lesser anti-folate properties were searched. It is similar in structure to drugs that inhibit dihydrofolate reductase (DHFR).

It is approved for adjunctive treatment and for cross-over to monotherapy for partial-onset and secondarily generalized tonic-clonic seizures, for LGS, and as add-on-therapy for primary generalized tonic clonic seizures in patients ≥ 2 years of ages. It is effective as a first line and adjunctive drug for generalized and focal seizures and may be an alternative to valproate in young women, because it does not provoke weight gain and ovarian problems. Its efficacy against PGTCSs parallels with carbamazepine.^7^ It may worsen myoclonic seizures in some patients with JME and LGS.

Studies showed that it is similarly effective in the treatment of focal epilepsy as CBZ or PHT, is better tolerated, has a favorable side effect profile when compared with VLP, has none or fewer adverse effects on sex hormones in women, and is likely to have a lower teratogenicity and lesser cognitive dysfunction.^8, 9^

Like PHT or CBZ, lamotrigine exerts most of its antiepileptic activity by blocking voltage-dependent Na-channels. It also reduces Ca^++^ currents and may have additional unknown effects, which may explain its broad spectrum of efficacy.

It is 55% protein-bound and has an elimination half-life of 24-40 hours (70 hours when combined with VPA). Lamotrigine is metabolized in the liver and excreted by kidneys, but neither induces nor inhibits hepatic enzymes. Therefore, has no effect on the serum levels of other AEDs and no dosing change is needed when is used concomitantly with OCPs or Warfarin. However, enzyme inducers may decrease lamotrigine half-life to 14 hours, requiring dose adjustment. At higher doses it may cause autoinduction. OCPs decrease LTG half-life, but as combined oral contraceptive monthly packs have seven days with no hormonal placebo pills, LTG level may raise by as much as 40%, leading to monthly fluctuations and causing side effects.

Lamotrigine is well tolerated if initiated slowly, but produces sleepiness and dizziness otherwise and during treatment with higher doses. Up to 5% of patients develop a rash, which is often associated with a rapid titration. A severe rash, more common in children on VPA, may develop and result in the rare and potentially fatal Steven-Johnson syndrome in 1% of cases.^10^ Other AEs include ataxia, diplopia, headache, tremor, blood dyscrasia, GI upset, psychosis, somnolence, insomnia, and milder hyper sensitivity reactions.

The usual initial dosage in adults is 12.5-25 mg/day. Maintenance dose is 200-600 mg/day, usually divided into two dosages. Dosage titration must be monitored particularly when LTG is given in combination with VPA, because the latter inhibits the hepatic metabolism of LTG. In contrast, target dosages of 600-1000 mg/day may be required to achieve therapeutic levels of LTG, when it is used together with enzyme inducers. It is prepared by 25, 50, 100, and 200 mg tablets.

### 4-Tiagabine (TGB: Gabitril)

Tiagabine is a derivative of the GABA uptake inhibitor, nipecotic acid, which reversibly inhibits GABA transporter-1.^11, 12^ In contrast to pregabalin and gabapentin, its protein binding is much higher (96%) and is not removed by hemodialysis. It is metabolized by the isoform 24 of the CYP 450 system in the liver, thus enzyme inducers markedly enhance its metabolism and increase its clearance.^13^ However, liver diseases reduce its removal from the body.

The elimination half-life of TGB is 7 to 9 hours. After multiple dosing, a steady plasma concentration is reached within 2 days. No clear relationship exists between TGB levels and clinical efficacy.

Tiagabine does not alter the efficacy of the oral contraceptives, warfarin, digoxin, or theophylline, but decreases valproate level by 10%. Cimetidine and VPA increase plasma level of TGB.

Tiagabine may induce convulsive and non-convulsive status epilepticus. Therefore, it should be used with caution in cases with a history of SE. This drug is contraindicated in absence of epilepsy and in partial seizures with generalized spike wave on EEG, where it may worsen seizure control.^12^

Its reported side effects include: tiredness, dizziness, confusion, nervousness, tremor, psychosis, flue-like symptoms, ataxia, word finding difficulty, and GI upset. It does not appear to have any effect on the visual filed, as vigabatrin did.

In adolescents of 12 to 18 years of age, it should be initiated at 4 mg once daily. The total daily dose may be increased by 4 mg at the beginning of the second week. Thereafter, the total daily dose may be increased 4 to 8 mg at weekly intervals until clinical response or maximum dose of 32 mg/day is achieved. In adults TGB should be initiated at 4 mg once daily. It may be increased by 4 to 8 mg at weekly intervals until the maximum dose of 50 mg per day. It is prepared as: 4, 12, 16, and 20 mg tablets.

### 5-Topiramate (TPR: Topamax)

Topiramate is a sulfamate-substituted monosaccharide with a broad spectrum activity that is currently approved for partial-onset seizures, SGTCS, LGS, and idiopathic generalized epilepsy in adults and children > 2 years of age.

It exerts its effect by: 1) blocking voltage-gated Na-channels, thus reducing duration of spontaneous bursts and the frequency of APs, 2) weak inhibition of carbonic anhydrase, 3) potentiation of GABA_A_ receptors, 4) inhibiting AMPA subtype glutamate receptor, and 5) reducing amplitude of high voltage-gated Ca-currents.^14^

Topiramate is licensed for use as monotherapy in partial seizures of adults in UK and other countries. It is better tolerated during monotherapy than polytherapy. The latter is often limited by its negative cognitive effects.^15^

Its bioavailability is 100%, has a protein binding of 15% and elimination half-life of 18-23 hours. The hepatic CYP 450 system metabolizes only 15% of the drug and the remainder is excreted unchanged in urine. Therefore, dose should be adjusted in patients with renal failure.

Topiramate has no effect on other drugs, except for increasing phenytoin levels (in contrast to vigabatrin that reduces the level of phenytoin). However, it decreases estradiol levels and may inactivate low-dose contraceptives at doses greater than 200 mg/day.^16^ Enzyme inducers decreased its level by 50% in some studies.

Paresthesia in the extremities, weight loss, hypohydrosis (especially in children), naming difficulty, speech slowing, confusion, memory and concentration impairment, diarrhea, diplopia, dizziness, fatigue, ataxia, depression, and agitation are the most common AEs. Increased risk of renal calculi, hyperchloremic non-anion gap metabolic acidosis, myopia in closed-angle glaucoma, and, rarely, an acute glaucoma are also reported. Generalized tonic-clonic seizure, acne, alopecia, and decreased level of hepatic enzymes have also been reported.

In adults, its initial starting dose is 25-50 mg/day that can be increased gradually. The maintenance dose of topiramate is 100-500 mg/day. It is prepared as 25, 100, and 200 mg tablets.

### 6- Levetiracetam (LEV: Keppra)

Levetiracetam was initially approved as add-on-therapy for partial and generalized seizures. It was approved by FDA as an adjunctive therapy in the treatment of partial onset seizures in adults and children with one month of age or older with epilepsy in 2012. It is also effective as add-on-therapy in patients with poorly controlled idiopathic generalized epilepsy with onset during adolescence, LGS, photosensitivity, and also as monotherapy.^32^

Its mechanism of action is not clearly understood. It is believed to interfere with release of the neurotransmitters by binding to synaptic vesicle glycoprotein 2A (SV2). This protein is involved in synaptic vesicle docking and fusion. It has recently been reported that LEV has other mechanisms of action including reducing Ca-currents, reversing inhibition of GABA and glycine gated currents induced by negative allosteric modulators, and affecting K^+^ channels conductance.

LEV is absorbed rapidly and completely, thus it is not affected when taken with food. Its protein binding is < 10%. Peak plasma concentration occurs within 48 hours and bioavailability is nearly 100%. Its half-life is 6-8 hours (prolonged in elderly).

This drug neither undergoes hepatic metabolism, nor induces or inhibits CYP 450 system. Thus, it is devoid of interactions with other AEDs, OCPs, anticoagulants, or digoxin. Approximately 27% of LEV is metabolized via hydrolysis by a serine esterase enzyme in blood and other tissues and is excreted through the kidneys unchanged or as inactive metabolites.

The most common AEs include somnolence, asthenia, and dizziness. Upper respiratory infection, depression, vertigo, insomnia, amnesia, dyspepsia, diarrhea, anorexia, rash, and diplopia have also been reported. During long-term treatment there was a slightly higher incidence of psychiatric side effects.

Its starting oral dose is 500 mg twice daily, which can be increased up to 3000 mg/day. Intravenous administration was approved as adjunctive therapy for partial seizures as well as for status epilepticus (Table 3).

**Table 3:** Preparations and administration of intravenous LEV

Dose	Preparations	Volume of diluents	Infusion time
500 mg	5 ml (5 ml vial)	100 ml	15 minutes
1000 mg	10 ml (two 5 ml vials)	100 ml	15 minutes
1500 mg	15 ml (three 5 ml vials)	100 ml	15 minutes

It has been assigned to pregnancy category C, and at present a sufficient amount of data is not available to recommend during pregnancy or lactation. It is prepared as 250, 500, and 1000 mg tablets.

### 7-Oxcarbazepine (OXC)

Oxcarbazepine is a ketoanalogue of carbamazepine that exerts its antiepileptic activity through blockage of voltage-sensitive Na-channels. It also reduces presynaptic release of glutamate and has a different effect on the N-type Ca-channels.^17, 18^ This drug was produced with the goal of avoiding CBZ's auto-induction and drug interactions. In general it has similar potency to CBZ, but a more favorable side effect profile (with exception of hyponatremia). OXC is nearly completely absorbed, its peak plasma levels are reached in four hours, has a half-life of 8-10 hours, and its protein binding is 40%.

It is broken down into the active metabolite (monohydroxy derivative: MHD) without generating epoxide (a metabolite responsible for many AEs of CBZ).

OXC is better tolerated than CBZ and PHT and similarly well tolerated as VLP. There is no clear difference between these drugs in term of effectiveness.^19^ However, OXC has weaker enzyme inducing effects than CBZ; therefore, it affects sex hormone metabolism less than CBZ. It has shown that testosterone levels, which are reduced in temporal lobe epilepsy, decrease further by CBZ. It is possible that such negative effects could be avoided in patients receiving OXC.^20, 21^ Although, OXC has little enzyme-inducer effect, it can significantly affect OCPs and render them ineffective in doses > 1200 mg.

OXC and CBZ were used successfully with additive benefit. Patients can be switched directly from CBZ to OXC (using a ratio of 2:3 in patients receiving CBZ up to 1500 mg/day and of 1:1 for higher doses). Some physicians advocate a more gradual switch in patients taking more than one AED or taking more than 800 mg/day of CBZ.^22, 23^ Patients who are switched from CBZ to OXC may experience improvements in terms of tolerability and effectiveness. However, some retrospective studies have uncovered the exacerbation of seizures in juvenile generalized epilepsies in patients treated with OXC.^24^ It is contraindicated in patients with hypersensitivity, porphyria, and hepatic or renal dysfunction.

The most common reported AEs include headache, weight gain, somnolence, dizziness, rash, hyponatremia, GI upset, and alopecia. In patients allergic to CBZ, allergic cross-reaction with OXC will arise in 25% of cases. Hyponatremia is reported in 2.5% of adults, is uncommon in children < 17 years of age, and higher (7.4%) in elderly patients. It is generally mild and can be easily treated with fluid restriction. However, it may be problematic in older patients, multimorbid cases, or those with cardiac and renal diseases, in whom regular blood electrolyte estimations are indicated.^25, 26^

In general, OXC is better tolerated than CBZ and has less potential for drug interactions, but is not indicated in idiopathic generalized epilepsy syndromes (especially in absence seizures), which it may exacerbate.^27^ It is prepared as 150, 300, and 600 mg tablets.

### 8-Zonisamide (ZNS)

In contrast to rufinamide, zonisamide is a broad spectrum AED with an efficacy similar to topiramate. It was approved by the FDA in 2000 for adjunctive treatment in patients older than 12 years of age with partial seizures.

Its primary indications include refractory partial epilepsy and generalized epilepsy of all types, LGS, West syndrome, and progressive myoclonic epilepsy. It is licensed in Japan and Asia in children and adults, but in the USA only for refractory partial seizures in patients > 12 years of age.

It is absorbed rapidly and completely, and its protein binding is similar to LTG (55%). Approximately, 70% is metabolized in the liver by CYP 450 system, but does not induce hepatic enzymes. Therefore, it has little effect on other AEDs. Zonisamide has a long half-life (60 hours), thus can be used once daily. Enzyme inducers decrease its half-life to 24 to 46 hours.

Six mechanisms of action have been reported for ZNS.^28–30^

1) Acts primarily by blocking Na-channels, 2) inhibits carbonic anhydrase, 3) inactivates T-type Ca-channels (which makes it effective for myoclonus as found in juvenile myoclonic epilepsy), 4) mediates GABA inhibition, 5) facilitates dopaminergic and serotonergic transmissions, and 6) inhibits release of glutamate.

Adverse effects are generally similar to that of topiramate with the exception that it can also cause 5% weight gain, headache, tremor, and spontaneous abortion, and has a cross-reaction with sulfonamides, thus should not be used in patients with past history of allergy to sulfonamides. Renal calculi are observed in 1.5% of cases and its carbonic anhydrase-inhibiting effect may cause anhidrosis in children. Tinnitus, flue-like symptoms, and weight loss are also reported.

In adults, its usual daily dose is 200-600 mg. In children it is initiated with 2-4 mg/kg/day, maintained on 4-8 (maximum 12) mg/kg/day. It is prepared as 100 mg capsules.

### 9-Pregabalin (PGB: Lyrica)

PGB is approved for add-on-therapy in partial-onset seizures of adults. It has also been effective against refractory epilepsy and status epilepticus.^31–34^ Recent studies demonstrated its efficacy in generalized anxiety disorder with a consistent effect on psychic and somatic symptoms.^35^ Furthermore, in doses ranging from 150 to 300 mg/day it reduces pain scores by more than 50% in patients with post herpetic neuralgia.^36^ Similar to carbamazepine, phenytoin, or gabapentin, it has a beneficial effect on many central and peripheral neuropathic pains.^37^

Like Gabapentin, it binds alpha-2 delta subunit of Ca^++^ channels and has a short half-life (6 hours), but its bioavailability is higher than gabapentin (> 90% versus 60%). It is not metabolized, has no effect on liver enzymes, is excreted unchanged by the kidneys, and has no significant drug interaction. Adverse effects include dizziness, somnolence, ataxia, asthenia, fatigue, and diplopia, which are dose dependent and usually mild. Dose related weight gain has also been reported in 18% of cases. It is important to mention that, pregabalin is eliminated by kidneys. No significant serious toxicity was reported in patients with significant renal dysfunction (Creatinine clearance < 60), though dosage should be lowered. In adults, the initial and maintenance doses are 150 mg/day and 150-600 mg/day respectively, which are divided into two daily doses. It is prepared as 25, 50, 75, 100, 150, 200, and 300 mg tablets.

### 10-Rufinamide (RNM)

Rufinamide is a new-AED from triazole derivatives and is structurally unrelated to other marketed antiepileptic drugs. It was approved as an effective add-on-therapy in atonic seizures of LGS in 2010.^38^ Before rufinamide, only felbamate, topiramate, and lamotrigine had a specific indication for this syndrome. Its mechanism of action is not fully understood. However, in vitro studies have suggested that rufinamide modulates sodium channels, especially by prolongation of time spent in the inactive state of the channels.

Common adverse effects are headache, dizziness, fatigue, and GI distress. More unique for this drug is cardiac conduction disturbances with QT interval shortening, which is mild, but may increase risk of ventricular dysrhythmia.^39, 40^ For this reason it should be avoided in patients with familial short QT syndrome and used with caution in combination with drugs that shorten QT interval.

### 11-Lacosamide (LCM)

LCM was approved by the FDA in 2009 for partial seizures of adults. LCM and LEV are the only antiepileptic drugs with both oral and intravenous formulations that expand options for patients who are unable to receive oral medication.^41^ In contrast to LEV, any potential role of IV-LCM for use in status epilepticus is yet to be determined.

It is believed that LCM stops seizure by enhancing or prolonging selectively the slow inactivation of Na+ channels. This mechanism of action is quite different from that of others such as phenytoin, carbamazepine, valproate, lamotrigine, felbamate, topiramate, or rufinamide, which act to block sodium channels in the fast inactivated state.

Studies showed that doses higher than 400 mg led to more AEs than the beneficial effects, thus 400 mg is the maximum safe dosage. During intravenous administration, the infusion rate is 300 mg within 30 to 60 minutes.

Its most common side effects are ataxia (6%) and dizziness (25%). Syncope was reported in a trial in patients with diabetic neuropathy. A small but dose-dependent PR-interval prolongation has also been observed.^42^ Thus, a screening EKG is appropriate in patients with myocardial disease, heart failure, or those who take drugs affecting PR-interval.

### 12-Vigabatrin (VGB: Sabril)

Of all the new drugs, vigabatrin is the only one which has shown superior potency to conventional drugs. However, this evidence is limited to the use in West syndrome, for which Sabril is now the first choice. This drug is a derivative of GABA that binds irreversibly to GABA-T (GABA-transaminase) and increases CNS GABA by inhibition of its metabolism.^43–45^ Therefore, its pharmacological activity is determined by the resynthesis of GABA, which takes 6 days after drug discontinuation.^46^ It may also stimulate GABA release.

VGB was initially approved in Europe in 1989 and then by the FDA in 2009 only for infantile spasms and as add-on therapy for refractory partial seizures in adults when other treatments have failed. In other words, vigabatrin offers a new option for adults with disabling partial epilepsy who have not responded to available AEDs, and is an alternative to corticotropin in children with infantile spasms.

The reason for this limitation is that vigabatrin causes irreversible visual filed defects in 30% to 60% of patients, possibly by GABA mediated toxic effects on retina amacrine cells.^46–50^ This defect presents as bilateral concentric visual field constriction, ranging from mild to severe tunnel vision. Thus, a formal ophthalmologic assessment is required every 3 months, and treatment should be stopped if no clinical benefit is achieved after 3 months of therapy. The visual field defect can occur any time during the treatment, with estimated risk of developing of 8% per year.

It is absorbed rapidly and plasma peak concentrations are attained within 2 hours. Its protein binding (< 5%) and elimination half-life (5-9 hours) are similar to those of gabapentin and pregabalin. Approximately 75% of the drug is excreted by kidneys, requiring dose reduction in patients with renal insufficiency.

This drug and tiagabine have advantages of low toxicity and few known side effects. Drowsiness and fatigue are the most common adverse effects. Headache, vertigo, ataxia, agitation, irritability, weight gain, depression, confusion, and to a lesser extent tremor, psychosis, memory loss, and diplopia have also been reported.^51^ Repeated high doses caused alopecia in animals.^52^ Peripheral neuropathy and edema are other adverse affects. VGB interacts only with phenytoin and reduces its levels 16% to 33%.

The seizure-inducing or seizure increasing property of VGB, which may present during therapy, has been attributed to the increase of a cerebral amount of GABA that may exhibit excitatory or inhibitory neuronal effects.

In adults, its initial daily dose is 1000 mg/day in one or two doses, which can be increased by 500 mg weekly up to the usual dose of 2000-3000 mg/day. The maximum daily dose is 4000 mg. In children it is started with 20-40 mg/kg and may be increased up to maximum dose of 40-100 mg/kg/day. It is prepared as 500 mg tablets.

### 13-Clobazam (CBM)

Clobazam is a benzodiazepine, a family that includes also lorazepam, diazepam, midazolam, and clonazepam. It was recently approved in the USA as adjunctive treatment for LGS in patients 2 years or older. Its licensed indications include add-on-therapy for epilepsy without any restrictions by seizure type. It is not licensed for monotherapy.

Clobazam is unique among other benzodiazepines, because of relatively low tendency to produce sedation and possibly lower incidence of loss of therapeutic effect over time. The latter features render clobazam appropriate for long-term maintenance therapy. It acts by potentiation of GABA-ergic neurotransmission via binding to the GABA_A_ receptor.

Its usual daily dose is 10-20 mg; higher doses can be used up to 0.5 mg/kg/day. In children aged between 3 and 12 years the usual daily dose is 5-10 mg.

The most common adverse effects are tiredness and sedation that tend to be dose related. Metal flavor and bradycardia are also reported. Patients should not receive other sedatives or alcohol, and discontinuation must be gradual. The most common AEs, leading to discontinuation of clobazam included lethargy, somnolence, ataxia, aggression, fatigue, and insomnia. It is contraindicated in patients with respiratory depression, severe renal failure, myasthenia gravis, narrow angle glaucoma, and untreated open angle glaucoma. It is prepared as 10 mg tablets.

### 14-Ezogabine (EZG)

Ezogabine that was approved for use as add-on-therapy for partial epilepsy in 2013 is a unique AED because of its mechanism of action. It appears to enhance potassium currents mediated by a particular family of Ion channels known as KCNQ, located on the presynaptic membrane of the excitatory synapses. Activation of these channels is thought to reduce neuronal excitability. It may also potentiate GABA_A_ receptors.^53, 54^

In patients with partial-onset seizures compared to placebo, ezogabine reduced seizure frequency with 600 mg/day (divided into three doses) by 27%. Doses of 900 and 1200 mg/day were used, but had showed less effect.

Its most concerning AEs are urinary retention (in 2%) neuropsychiatric symptoms, somnolence, and dizziness. Half of the patients with urinary retention required catheterization and after withdrawal of ezogabine 1 of 14 cases needed ongoing, intermittent self-catheterization. Therefore, patients at high risk for urinary symptoms, particularly urinary obstruction should be carefully assessed. This is particularly true for patients with benign prostatic hypertrophy or those taking other drugs that can affect urination. Neuropsychiatric symptoms (confusion and hallucinations) were frequent, but resolved rapidly following discontinuation of ezogabine.^55^ QT prolongation has also been detected in healthy volunteers taking 1200 mg/day ezogabine. This drug has a potential for abuse and dependence thus is classified by the FDA as a controlled substance. Caution should also be used in patients with preexisting cardiac conduction abnormalities or using medications known to increase QT intervals.

Ezogabin may increase digoxin levels and can induce its toxicity. Ethanol increases its levels, but CBZ and PHT decrease levels of ezogabine by 31 to 34%.
